# Sequence variation at the *MTHFD1L-AKAP12* and *FOPNL* loci does not influence multiple myeloma survival in Sweden

**DOI:** 10.1038/s41408-019-0222-8

**Published:** 2019-07-30

**Authors:** Mina Ali, Konstantinos Lemonakis, Anna-Karin Wihlborg, Ljupco Veskovski, Ingemar Turesson, Ulf-Henrik Mellqvist, Urban Gullberg, Markus Hansson, Björn Nilsson

**Affiliations:** 1 Hematology and Transfusion Medicine, Department of Laboratory Medicine, BMC B13, 221 84 Lund, Sweden; 2 Hematology Clinic, Skåne University Hospital, 221 85 Lund, Sweden; 3 Section of Hematology, South Elvsborg Hospital, SE 501 83 Borås, Sweden; 4 Wallenberg Center for Molecular Medicine, 221 84 Lund, Sweden; 5 Broad Institute, 7 Cambridge Center, Cambridge, MA 02142 USA

**Keywords:** Myeloma, Cancer genetics

Multiple myeloma (MM) is the second most common hematologic malignancy. The disease is defined by an uninhibited, clonal growth of plasma cells in the bone marrow^1^. It is preceded by monoclonal gammopathy of unknown significance (MGUS)^2^, a common condition defined as a clonal growth of plasma cells that does not yet satisfy the criteria for MM, but progresses to MM at a rate of ~1% per year^3^.

Increasing evidence supports that the biology of MM is influenced by inborn genetic variation. MM and MGUS show familial clustering, and genome-wide association studies have identified DNA sequence variants that influence MM risk^4–8^. Additionally, two recent studies indicate that genetic variation could also influence MM survival^9,10^.

In the first of these, Johnson et al.^9^ describe an association between overall survival in multiple myeloma (MM-OS) and rs12374648, located between the *MTHFD1L* and *AKAP12* genes at chromosome 6q25.1^9^. The protein encoded by *MTHFD1L* is involved in folate metabolism^11^, and *AKAP12* is related to cell growth^12^. The association with MM-OS was detected in a meta-analysis of 3256 cases from four clinical trials: two from the UK, one from the USA, and one from Germany (combined *P*-value = 4.69 × 10^–9^, hazard ratio, HR = 1.34, 95% CI 1.22–1.48). The association was present in the sample sets from the UK and USA (*P* = 1.69 × 10^–6^ to 0.009; HR 1.06 to 1.75), but not in the one from Germany (*P* = 0.55; HR = 1.09, 95% CI 0.82–1.44). No replication in independent material was carried out after the discovery meta-analysis.

In a second study, Ziv et al.^10^ describe an association between MM-OS and rs72773978 near *FOPNL* at 16p13. The protein encoded by *FOPNL* has been implicated in centrosome function^13^. The association was detected by meta-analysis of 545 cases from two clinical trials in USA (*P* = 6 × 10^–10^; HR = 2.65, 95% CI 1.94–3.58). The association was also seen in a meta-analysis of seven other data sets (six from the IMMEnSE study totaling *n* = 772 and one from Utah, *n* = 315) (combined *P* = 0.044; HR = 1.34, 95% CI 1.01–1.78). Yet, the positive replication result was driven by a *P*-value of 0.004 with unrealistically large effect size (HR = 9.73) in a subset of 109 patients from Spain in IMMEnSE, whereas the other six subsets (Italy, Poland, Portugal, Denmark, Edmonton in IMMEnSE and the one from Utah) did not show any evidence of association (Supplementary Table 7 in ref. ^10^).

Given that the *MTHFD1L* association was not replicated after discovery analysis and that the *FOPLN* association was based on small sample sizes, it remains a possibility that these two associations are false discoveries due to a winners curse effect. We therefore looked for further support of the *MTHFDL1-AKAP12* and *FOPLN* loci in a Swedish study population. For this, we retrieved clinical data for 871 patients diagnosed with MM between 2005 and 2015 from the Swedish Multiple Myeloma Registry (Sahlgrenska Hospital, Gothenburg) (Table 1), which records clinical data on MM patients in Sweden and has about 90% inclusion rate compared to the Swedish Cancer Registry. The patients had been previously genotyped in genome-wide association studies using population-based samples from the Swedish National Myeloma Biobank (Skåne University Hospital, Lund)^6,7^. The clinical data and samples were obtained subject to informed consent and ethical approval (Lund University, dnr 2013/540), and in accordance with the principles of the Declaration of Helsinki. The samples were genotyped using Illumina microarrays and imputed with phased reference haplotypes from 1000 Genomes^6,14^. To test for association between genotypes and MM-OS, we used log rank test implemented in R (v.2.8) with adjustment for age, sex, and International Staging System (ISS) score. Survival was calculated from the date treatment started until the date of death, or until 5 April 2016 (median follow-up time 39.5 months).

**Table 1:** Clinical characteristics of the study population

Number of cases	871
Gender	
Male	531
Female	340
Median age at diagnosis	68
Median follow-up (months)	39.48
Deceased during follow-up	
Yes	393
No	478
ISS	
I	179
II	339
III	234
Unknown	119
Heavy chain paraprotein	
IgA	191
IgG	536
IgD	6
IgM	6
Not detected	132
Light chain paraprotein	
Lambda	240
Kappa	446
Not detected or not done	185
Median plasma cells in bone marrow (%)	22
Treatment received	
Proteasome inhibitor	427 (49.02%)
Immunomodulatory (IMiD)	228 (26.18%)
Chemotherapy	678 (77.84%)
Autologous stem cell transplantation (ASCT)	283 (32.49%)
Other or no treatment	112 (12.86%)
Anemia (%)	26.18
Hypercalcemia (%)	8.04
Renal failure (%)	13.6

**Fig. 1:**
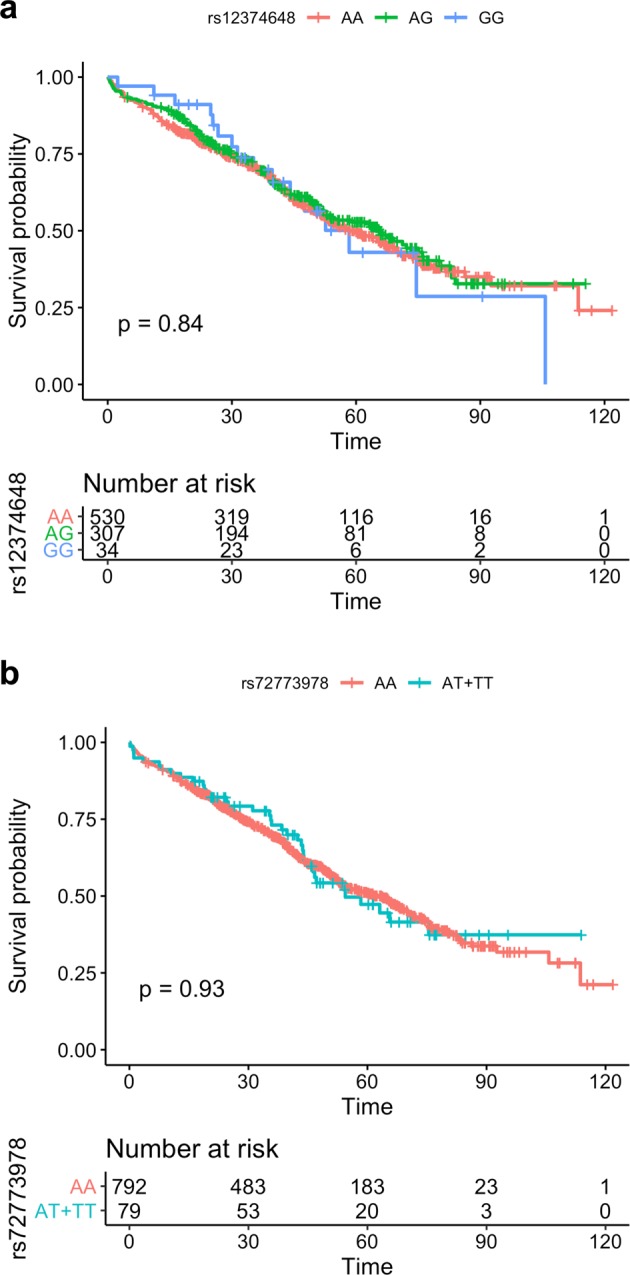
Kaplan-Meier plots for **a** rs12374648 at *MTHFD1L-AKAP12* and **b** rs72773978 at *FOPNL*. No difference in survival between the genotype groups was observed

In our analysis, we did not see any evidence of association with MM-OS for either rs12374648 (*P* = 0.84; HR = 0.97, 95% CI = 0.81–1.2) or rs72773978 (*P* = 0.93; HR = 0.98, 95% CI = 0.7–1.4) (Fig. 1). For completeness, we also tested for associations between MM-OS and all variants with minor allele frequency (MAF) >5% located within 1 Mb of *MTHFD1L-AKAP12* (6,515 variants) or *FOPNL* (3,892 variants) but could not identify any significant association with any of these variants. Thus, we could not replicate the associations between MM-OS and *MTHFD1L-AKAP12* and *FOPNL* in a population-based series, nor identify any other alleles associations with MM-OS at these loci.

Our results, in conjunction with the small sample sizes, lack of robust replication in the original studies, and the fact that the original studies do not replicate each other, indicate that the reported associations are false positives. As for alternative explanations, a first possibility could be limited power of our data set. Yet, our sample is comparable in size (*n* = 871) to the largest of the reported individual sample sets, including UK-My9 (*n* = 1,163) and UK-My11 (*n* = 871) where rs12374648 at *MTHFD1L* was detected, and substantially larger than the data sets where rs72773978 at *FOPNL* was detected. Power calculations^15,16^ indicate that our sample set has about 71% chance to detect an effect with HR = 1.34 (the effect size of rs12374648 and the replication effect size of r72773978), and about 99% chance to detect an effect with HR = 2.65 (the discovery effect size of rs72773978), in our sample set. A second possibility could be differences in geographic origin. However, this also seems unlikely given that the two reported variants are common, both in our data (MAF 21.5 and 4.7%) and in the different populations of 1000 Genomes^14^. Finally, a third possibility could be differences in clinical characteristics between the study populations. One difference is that our material is population-based, whereas the studies by Johnsson et al.^9^ and Ziv et al.^10^ are based on patients recruited into clinical trials. As a result, our population is older (average 68 years vs 54–66 years), and has not been selected for patients without comorbidity, as is common in clinical trials. A higher incidence of comorbidity could dilute effects of DNA sequence variation on survival, and differences in age and comorbidity will carry differences in treatment. For example, some of the reported populations contain a high proportion of patients who received autologous stem cell transplantation (ASCT; 100% in the German and US sample sets in Johnson et al.^9^), whereas our study population contains 32.5% transplanted patients.

In summary, our results together with the limitations of the original studies indicate that the reported associations between the *MTHFD1L* and *FOPNL* loci and MM survival are false positives due to a winner’s curse effect. While there could be alternative explanations, these seem unlikely in comparison. Our results motivate the collection of larger data sets to understand the impact of genetic variation on clinical outcome in MM.
